# Dexmedetomidine Pretreatment Attenuates Kidney Injury and Oxidative Stress during Orthotopic Autologous Liver Transplantation in Rats

**DOI:** 10.1155/2016/4675817

**Published:** 2015-11-22

**Authors:** Xiaofang Yu, Xinjin Chi, Shan Wu, Yi Jin, Hui Yao, Yiheng Wang, Zhengyuan Xia, Jun Cai

**Affiliations:** ^1^Department of Anesthesiology, Third Affiliated Hospital, Sun Yat-sen University, Guangzhou, Guangdong 510630, China; ^2^Department of Anesthesiology, Fujian Provincial Hospital, Fuzhou, Fujian 350001, China; ^3^Department of Pathology, Third Affiliated Hospital, Sun Yat-sen University, Guangzhou, Guangdong 510630, China; ^4^Department of Anesthesiology, First Affiliated Hospital, University of South China, Hengyang, Hunan 421001, China; ^5^Department of Anesthesiology, The Second Affiliated Hospital & Yuying Children's Hospital of Wenzhou Medical University, Wenzhou, Zhejiang 325027, China

## Abstract

This paper aims to explore whether pretreatment with dexmedetomidine (Dex) has antioxidative and renal protective effects during orthotopic autologous liver transplantation (OALT) and its impact on nuclear factor erythroid 2-related factor 2 (Nrf2) activation. Sprague-Dawley rats were randomized into groups that include sham-operated (group S), model (group M), low dose Dex (group D1), high dose Dex (group D2), atipamezole (a nonspecific *α*_2_ receptor blocker) + high dose Dex (group B1), ARC239 (a specific *α*_2B/c_ receptor blocker) + high dose Dex (group B2), and BRL-44408 (a specific *α*_2A_ receptor blocker) + high dose Dex (group B3). Then histopathologic examination of the kidneys and measurement of renal function, the renal Nrf2 protein expression, and oxidants and antioxidants were performed 8 hours after OALT. We found that pretreatment with Dex activated Nrf2 in glomerular cells and upregulated antioxidants but reduced oxidants (all *P* < 0.01, group D2 versus group M). Atipamezole and BRL-44408, but not ARC239, reversed these protective effects. In conclusion, pretreatment with Dex activates Nrf2 through *α*_2A_ receptor, increases the antioxidant levels, and attenuates renal injury during OALT.

## 1. Introduction

Orthotopic liver transplantation (OLT) has been considered the best choice for end-stage liver diseases [1]. Acute kidney injury (AKI) is the most common and severe complication after OLT with a 12%–70% [2, 3] incidence rate and an annual mortality rate of up to 35%–45% [4]. It is an important factor in early postoperative death and influences the prognosis and rehabilitation of patients [5, 6]. Therefore, it is important to try to increase the survival rate and improve the rehabilitation after OLT by means of controlling and/or avoiding acute kidney injury in the perioperative period of liver transplantation. Previous studies have demonstrated that the oxidative stress injury is related to AKI after OLT.

Nuclear factor erythroid 2-related factor 2 (Nrf2) is a master transcriptional factor in cells, which acts against oxidative and stress injury. It can enhance the antioxidant enzymes and phase II detoxification enzyme levels in combination with the antioxidant response element (ARE) and plays a key role in the endogenous antioxidative activity [7, 8]. Recent studies have borne out claims that it plays an important role in preventing ischemia-reperfusion injury. Some researchers have reported that Nrf2 can provide protective effect by upregulating the expression of heme oxygenase 1 (HO-1), nicotinamide-adenine dinucleotide phosphate (NADPH), glutathione reductase (GR), and glutathione peroxidase (GPx). But whether the AKI after OLT is associated with Nrf2 is still uncertain [9–12].

Dexmedetomidine (Dex) is a highly selective alpha 2 adrenergic receptor (*α*_2_-AR) agonist. Because of its effect on alpha 2 receptors of the locus ceruleus, Dex can provide an ideal sedative and analgesic [13–15]. Recently, many studies have found that Dex protects the heart, brain, and small bowel. Bell et al. had reported that Dex offers good perioperative hemodynamic stability and neuroprotective effects [16]. Wanga et al. demonstrated that perioperative treatment with propofol and Dex conferred neuroprotection against I/R injury in rats, and this protective effect was correlated with the antioxidant, anti-inflammatory, and antiapoptotic properties of propofol and/or Dex through a significant reduction in free radical release and production of proinflammatory cytokines, as well as acute reversal of activation of the apoptosis-related proteins [17].

Those researches about Dex and AKI after OLT are rare; in particular, no one had reported that the subtype of *α*_2_-AR is related with the protection of the renal function. The aim of the study was to determine whether pretreatment with an *α*_2_ adrenergic receptor activator, Dex, has antioxidative protective renal effects mediated by Nrf2 during orthotopic autologous liver transplantation (OALT) in a rat model.

## 2. Materials and Methods

### 2.1. Animals

Male Sprague-Dawley (SD) rats, aged 8–10 weeks, weighing 220–250 g, were purchased from the Medical Experimental Animal Center of Guangdong Province. This study was performed with the approval of the Institutional Animal Care and Use Committee of Sun Yat-sen University in Guangzhou, China, and in accordance with the principles stated in the Guide for the Care and Use of Laboratory Animals (National Institutes of Health, 1985). All rats were housed in individual cages in a temperature-controlled room with alternating 12 h light/dark cycles. The rats were acclimated for one week prior to experiments. Food was withheld 12 h before the start of experiments, but all animals had free access to water.

### 2.2. Experimental Groups

Fifty-six rats weighing 220–280 g, fed by animal feed and raised in 25–27°C, were randomly divided into 7 groups with 8 rats/group. Sham-operated group (S) did not undergo I/R. Model group (M) was pretreated with normal saline by i.p. injection 30 min before operation. Rats in group D1 received 10 *μ*g/kg Dex (Hengrui Pharmaceutical Co., Ltd., Jiangsu, China) by i.p. injection 30 min before operation. Rats in group D2 received 50 *μ*g/kg Dex by i.p. injection 30 min before operation. Rats in groups B1, B2, and B3 received 500 *μ*g/kg atipamezole (a nonspecific *α*_2_ receptor blocker, Sigma-Aldrich, USA), 50 *μ*g/kg ARC239 (a specific *α*_2B/c_ receptor blocker, Santa Cruz, USA), and 1.5 mg/kg BRL-44408 (a specific *α*_2A_ receptor blocker, Sigma-Aldrich, USA) by i.p. injection 40 min before receiving 50 *μ*g/kg Dex prior to OALT.

### 2.3. Construction of Rat OALT Model

A standard model of OALT was created as previously described [18, 19]. The rats were fasted for 12 h and anesthetized using 2% inhaled isoflurane (Baxter Healthcare Corporation), which were administered through an open face guard, and fixed on the electric blanket. Following the anesthesia, the abdomen was incised, exposing the manubrium and xiphoid ensistermum. Then we isolated the liver in anticlockwise direction, the left triangular ligament of the liver was resected and the left phrenic vein was ligated and disconnected, and we covered the liver with a wet gauze, opened the retroperitoneum, and isolated the bare area of liver. The suprahepatic vena cava (SVC) was fully liberated, and the right suprarenal vein was ligated. Once the liver was replaced in its original position, the inferior vena cava (IVC) was dissociated, the first hepatic portal was exposed, and the portal vein (PV) was separated from the splenic veins and the convergence of the inferior mesenteric vein. The hepatic artery and the biliary tract were liberated together according to their anatomic relationship. Before ischemia, we injected 1 mL heparin saline solution through the caudal vein. Subsequently, the microvascular clamps were folded on the convergence of the inferior mesenteric vein, hepatic artery, splenic vein, SVC, and IVC. The PV was punctured with a 4th needle in preparation for reperfusion and a 1 mm incision was made in the wall of the IVC as an outflow tract. Ringer lactate solution (precooled, 0~4°C) was injected during reperfusion at a speed of 2.0 mL/min and we cooled the liver with iced physiological saline at the same time until the liver color progressively turned yellow which meant the reperfusion was successful. Finally, after the needle was extracted, the incision of the PV was closed using 9-0 sutures and the opening of IVC was repaired by 8-0 sutures. The clamps on the PV, SVC, IVC, and hepatic artery were loosened and we rewarmed the liver with warm saline in the meanwhile. The whole anhepatic phase lasted for 20 ± 1 min. We have determined previously that kidney damage was most severe and the level of oxidative stress highest 8 h after reperfusion, during OALT [19]. For this reason, the 8 h time point was selected for the current experiments.

### 2.4. Collection of the Specimens

Rats received inhalational 1-2% isoflurane (Baxter Healthcare Corporation) 8 h after OLT during which 1 mL of blood was obtained by cardiac puncture. The sample was centrifuged for 10 min at 1500 r/min and the supernatant stored at −20°C. The left kidney was excised and fixed in 4% paraformaldehyde and prepared for pathological and immunofluorescence analyses.

### 2.5. Examination of the Renal Histopathology [19]

The kidney specimens were embedded in paraffin, cut into 5 *μ*m sections, stained with hematoxylin and eosin staining for microscopic examination, and scored on the pathologic change. The pathologist was blinded to the protocol.

### 2.6. Assessment of Kidney Function

Serum creatinine (Scr) and blood urea nitrogen (BUN) were test in blood sample with automatic biochemistry analyzer (Olympus AU640).

### 2.7. The Expression of Nrf2 by Immunofluorescence Staining

After dewaxing, samples were heated and then incubated with 3% hydrogen peroxide. Rabbit monoclonal anti-Nrf2 antibody (Abcam, UK) was added to the slices and incubated at 4°C overnight; FITC-labeled goat anti-rabbit IgG antibody was added for 2 h and mounted with antifluorescence quenching coverslip which contained DAPI. Imaging was performed with a Zeiss LSM 510 confocal microscopic system [20]. The fluorescence intensity was determined by Image-Pro Plus version 5.0 system (Media Cybemetics, USA).

### 2.8. Measurement of Oxidative Indexes, Such as Superoxide Dismutase (SOD), Glutathione (GSH), Total Antioxidant Capacity (T-AOC), Hydrogen Peroxide (H_2_O_2_), Hydroxyl Free Radical (∙OH), and Malondialdehyde (MDA)

The contents of SOD, GSH, T-AOC, H_2_O_2_, ∙OH, and MDA in renal tissue were measured by assay kits (Kaiji Biological Technology Development Co., Ltd., Nanjing, China).

### 2.9. Statistical Analysis

Statistical analysis was performed by using SPSS 12.0 software. Quantitative data were presented as mean ± SEM. Multiple comparisons among groups were analyzed by one-way ANOVA. The paired comparisons were using Fisher's Least Significant Difference (LSD) Procedure. All statistical tests were two-sided, and *P* < 0.05 were considered to be statistically significant.

## 3. Results

### 3.1. Characteristics of Rats among Groups

The weight of rats and the time of anhepatic phase among 7 groups were similar, as shown in Table 1.

### 3.2. Remote Kidney Damage in the Groups

**Figure 1:**
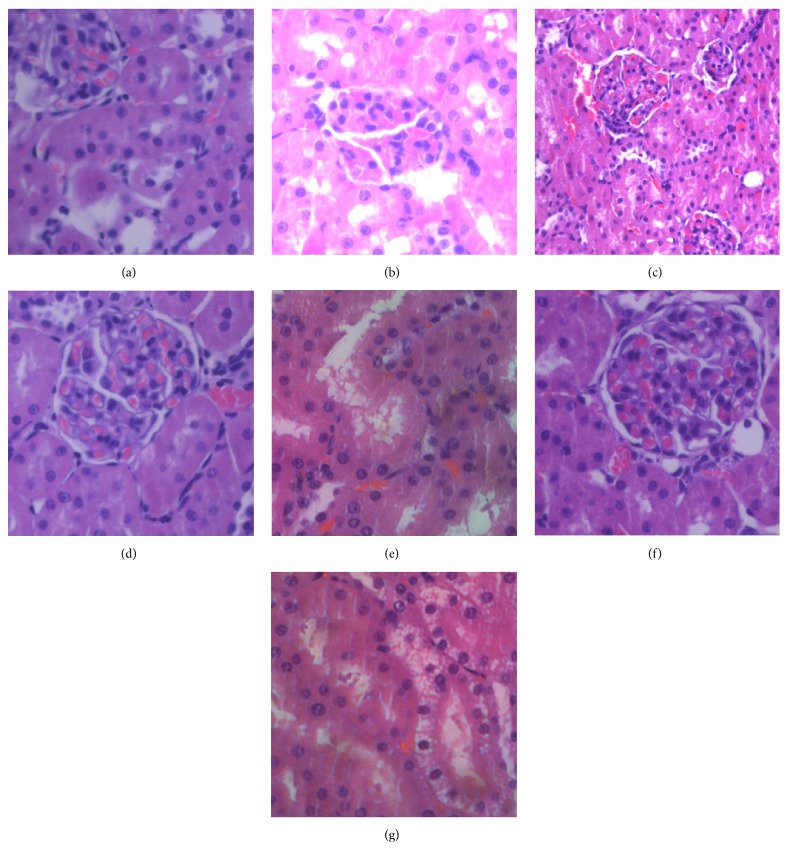
Remote kidney damage of rats in various groups after OALT. Kidney sections were stained with hematoxylin and eosin (HE) and visualized at ×400 magnification. (a) Group S; (b) group M; (c) group D1; (d) group D2; (e) group B1; (f) group B2; and (g) group B3.

**Figure 2:**
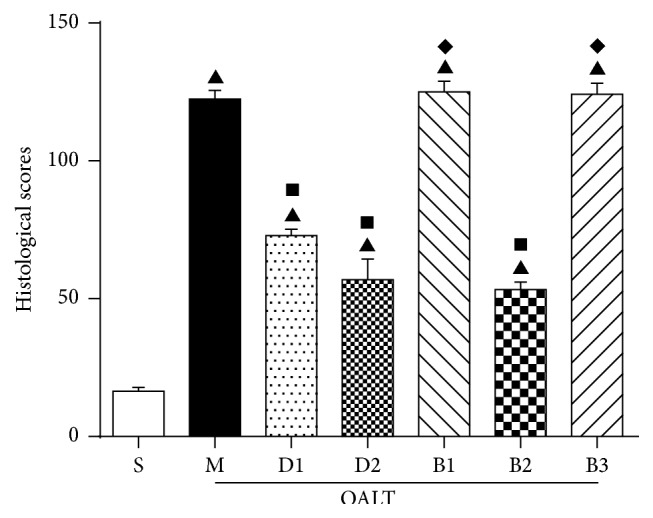
Pathological scores of kidney tissue. A score from 0 to 3 was given for each tubular profile: 0 = normal histology; 1 = mild injury: tubular cell dilatation (expanded 50 times its normal size); 2 = moderate injury: epithelial cells which are flattened, moderate tubular dilatation, or partial nuclear condensation; 3 = severe injury: the shape of healthy cells lost, with severe tubular dilatation or nuclear condensation. The data were represented as mean ± standard deviation (SD), *n* = 8. ^▲^ *P* < 0.01 versus group S; ^■^ *P* < 0.01 versus group M; ^*◆*^ *P* < 0.01 versus group D2.

As shown in Figures 1 and 2, compared with group S, the kidney damage in group M was severe (16.38 ± 4.21 versus 122.38 ± 8.99, *P* < 0.01) and included renal tubular cavity expansion and flat renal tubular epithelial cells. Missing nuclear staining could be obviously observed under light microscope. Compared with group M, the pathological changes and scores of rats pretreated with Dex, groups D1 (122.38 ± 8.99 versus 7.88 ± 6.53, *P* < 0.01) and D2 (122.38 ± 8.99 versus 56.75 ± 9.71, *P* < 0.01), were significantly lower, especially in group D2 which received 50 *μ*g/kg Dex. Compared to group D2, the protective effect was reversed in group B1 (56.75 ± 9.71 versus 125 ± 11.19, *P* < 0.01) which received atipamezole and in group B3 (56.75 ± 9.71 versus 124.13 ± 11.36, *P* < 0.01) which received BRL-44408. There was no effect in group B2 (56.75 ± 9.71 versus 53.25 ± 7.89, *P* > 0.05).

### 3.3. Changes of Serum Creatinine and Blood Urea Nitrogen Levels

**Figure 3:**
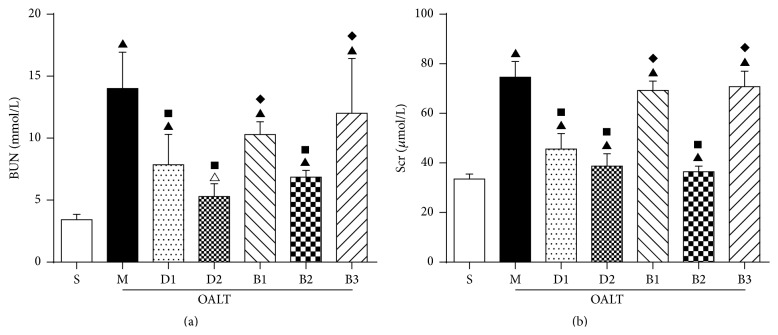
Levels of Scr and BUN at various groups. (The blood samples were taken from the abdominal aorta 8 h after reperfusion. The data were represented as mean ± standard deviation (SD), *n* = 8. ^▲^ *P* < 0.01, ^△^ *P* < 0.05 versus group S; ^■^ *P* < 0.01 versus group M; ^*◆*^ *P* < 0.01 versus group D2.)

As shown in Figure 3, compared with group S, the values of Scr (Figure 3(b)) and BUN (Figure 3(a)) increased significantly in group M (33.50 ± 5.86 *μ*mol/L versus 74.63 ± 17.89 *μ*mol/L, *P* < 0.01, and 3.43 ± 1.13 mmol/L versus 14.00 ± 7.77 mmol/L, *P* < 0.01). Compared with group M, the levels of Scr and BUN were decreased significantly in D1 (74.63 ± 17.89 *μ*mol/L versus 45.63 ± 17.73 *μ*mol/L, *P* < 0.01, and 14.00 ± 7.77 mmol/L versus 7.86 ± 6.47 mmol/L, *P* < 0.01) and D2 (74.63 ± 17.89 *μ*mol/L versus 38.69 ± 14.22 *μ*mol/L, *P* < 0.01, and 14.00 ± 7.77 mmol/L versus 5.29 ± 2.75 mmol/L, *P* < 0.01). Compared to group D2, the values of Scr and BUN increased significantly in group B1 (74.63 ± 17.89 *μ*mol/L versus 69.25 ± 10.66 *μ*mol/L, *P* < 0.01, and 14.00 ± 7.77 mmol/L versus 10.29 ± 2.75, *P* < 0.01) and group B3 (74.63 ± 17.89 *μ*mol/L versus 70.75 ± 38.69 *μ*mol/L, *P* < 0.01, and 14.00 ± 7.77 mmol/L versus 12 ± 11.74 mmol/L, *P* < 0.01). This protective effect was reversed in group B1 which received atipamezole and group B3 which received BRL-44408. There was no effect in group B2 (74.63 ± 17.89 *μ*mol/L versus 36.45 ± 6.37 *μ*mol/L, *P* > 0.05, and 14.00 ± 7.77 mmol/L versus 6.86 ± 1.46 mmol/L, *P* > 0.05).

### 3.4. Nrf2 Immunofluorescence Staining and Analyzing

**Figure 4:**
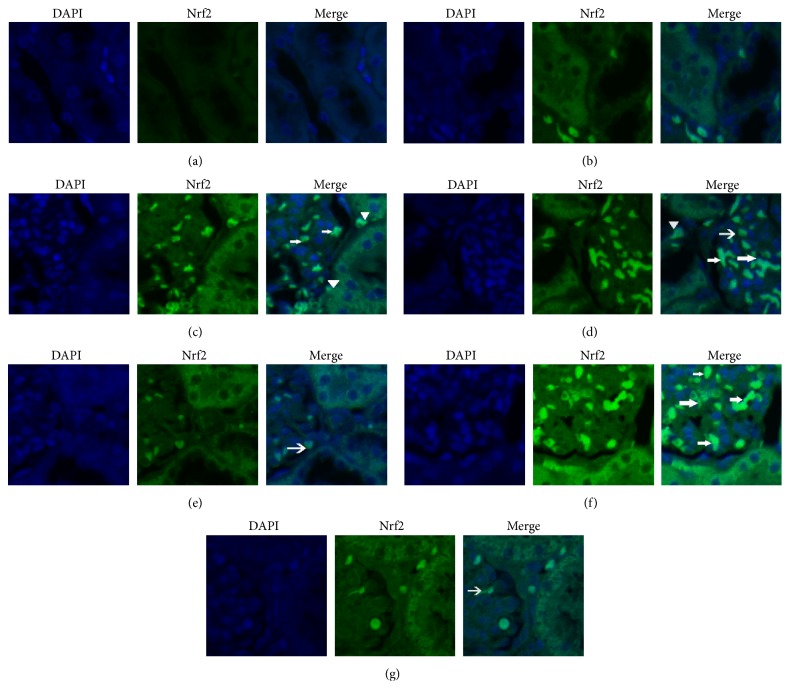
The expression of Nrf2 in kidney tissues of different groups was detected by immunofluorescence staining under a laser scanning confocal microscope (magnification 400x). (a) Group S; (b) group M; (c) group D1; (d) group D2; (e) group B1; (f) group B2; and (g) group B3. Positive Nrf2 cells were stained green, with the sections counterstained with 4,6-diamidino-2-phenylindole (DAPI) to visualize nuclei. Arrows indicate Nrf2 positive glomerular cells, while the triangle points at Nrf2 positive tubular epithelial cells.

**Figure 5:**
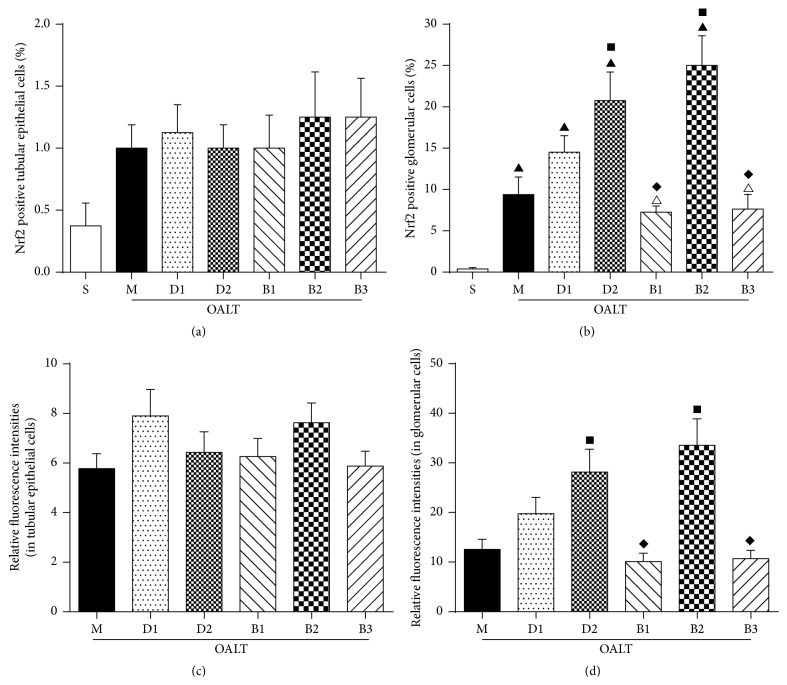
The proportion of Nrf2 positive cells and semiquantitative immunofluorescence of Nrf2 in glomerular cells or tubular epithelial cells in kidney tissues of different groups. The cells were counted under a laser scanning confocal microscope (magnification 400x). (a) The proportion of Nrf2 positive cells in tubular epithelial cells; (b) the proportion of Nrf2 positive cells in glomerular cells; (c) semiquantitative immunofluorescence of Nrf2 in tubular epithelial cells; (d) semiquantitative immunofluorescence of Nrf2 in glomerular cells. Relative immunofluorescence intensities were normalized to that of group S. The data were represented as mean ± standard deviation (SD), *n* = 8. ^▲^ *P* < 0.01 versus group S; ^■^ *P* < 0.05 versus group M; ^*◆*^ *P* < 0.05 versus group D2.

Nrf2 is a transcriptional factor which has been shown to have a protective effect by decreasing oxidative and stress injury. Increases in nuclear Nrf2 are associated with an improvement in levels of antioxidative and antistress factors of cells, resulting in less damage. Immunofluorescence intensities with positive Nrf2 protein expression as detected by Image-Pro Plus system are in general in accordance with the quantity/trend of protein expression measured by WB as previously reported [21–23]. Therefore, we also analyzed the immunofluorescence intensities of Nrf2 protein expression/staining in glomerular cells and in tubular epithelial cells, respectively. As shown in Figures 4 and 5, compared with group M, the expression of Nrf2 in glomerular cells in groups D1 and D2 was increased, especially in group D2. Compared with group D2, levels were lower in groups B1 and B3, while, in group B2, there was no obvious decrease.

### 3.5. Changes of Antioxidants and Oxidants

SOD is an enzyme which can catalyze breakdown of superoxidants into oxygen and hydrogen peroxide. GSH can convert hydrogen peroxide into oxygen and water. Both are the important antioxidants in the body. MDA is an end-product of lipid peroxidation in cell membranes. ∙OH is one of the most active free radicals. H_2_O_2_ is a strong oxidant. All can damage the cells, leading to cell death or mutations.

#### 3.5.1. Changes of Activity of SOD

**Figure 6:**
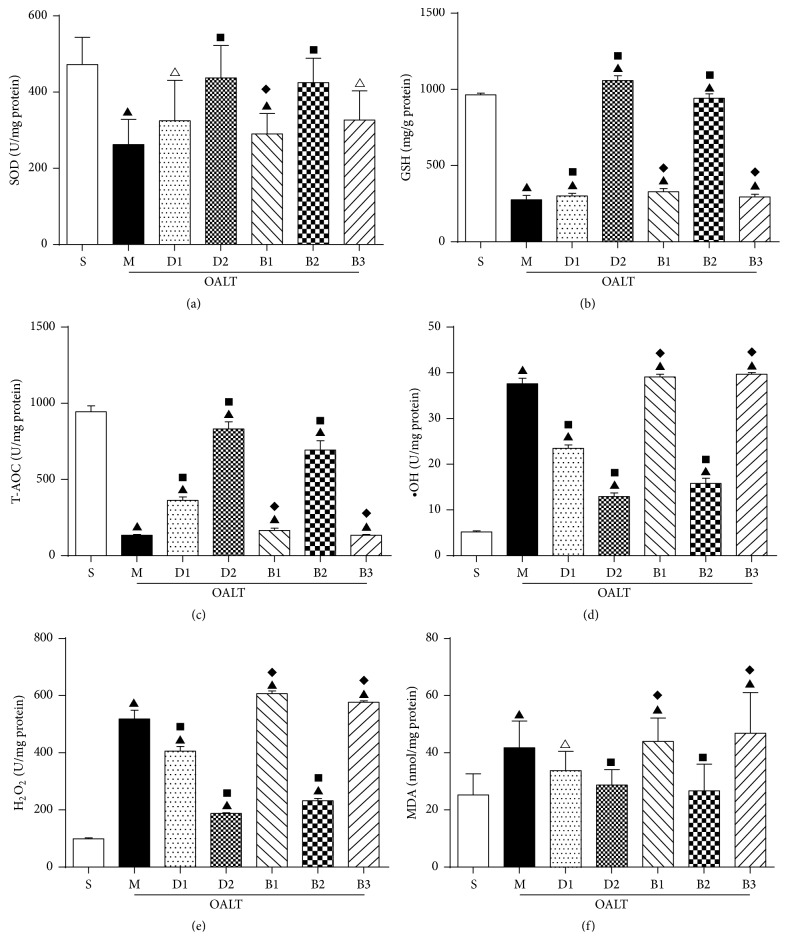
The antioxidants and oxidants of renal tissue. The data were represented as mean ± standard deviation (SD), *n* = 8. ^▲^ *P* < 0.01, ^△^ *P* < 0.05 versus group S; ^■^ *P* < 0.01 versus group M; ^*◆*^ *P* < 0.01 versus group D2.

As shown in Figure 6(a), the activity of SOD decreased in group M compared to group S (*P* < 0.01). In groups pretreated with high dose of Dex (group D2) and ARC239 (group B2), SOD increased significantly compared to group M (*P* < 0.01), while there was no significant difference in groups D1, B1, and B3 (*P* > 0.05).

#### 3.5.2. Changes of Activity of GSH

As shown in Figure 6(b), the activity of GSH decreased in group M compared to group S (*P* < 0.01). In groups pretreated with Dex (groups D1, D2) and ARC239 (group B2), GSH increased significantly compared to group M (*P* < 0.01), while there was no significant difference in groups D1, B1, and B3 (*P* > 0.05), compared to group D2. The level of GSH decreased significantly in group B1 and group B3 (*P* < 0.01). In the kidney, Nrf2 activation has been shown to play important roles in the maintenance of GSH levels [24, 25]. However, whether or not and how Nrf2 activation through its targets may regulate GSH have been largely unknown, which may deserve further study.

#### 3.5.3. Changes of Activity of T-AOC

As shown in Figure 6(c), the activity of T-AOC decreased significantly in all groups compared to group S (*P* < 0.01). In groups pretreated with Dex (groups D1, D2) and ARC239 (group B2), it increased significantly compared to group M (*P* < 0.01). There was no significant difference in groups D1, B1, and B3 (*P* > 0.05), compared to group D2. The level of T-AOC decreased significantly in group B1 and group B3 (*P* < 0.01).

#### 3.5.4. Changes of Activity of ∙OH

As shown in Figure 6(d), the activity of ∙OH increased significantly in all groups compared to group S (*P* < 0.01). In groups pretreated with Dex (groups D1, D2) and ARC239 (group B2), ∙OH decreased significantly compared to group M (*P* < 0.01). There was no significant difference in groups D1, B1, and B3 (*P* > 0.05), compared to group D2. The level of  ∙OH increased significantly in group B1 and group B3 (*P* < 0.01).

#### 3.5.5. Changes of Activity of H_2_O_2_

As shown in Figure 6(e), the activity of H_2_O_2_ increased significantly in all groups compared to group S (*P* < 0.01). In groups pretreated with Dex (groups D1, D2) and ARC239 (group B2), H_2_O_2_ decreased significantly compared to group M (*P* < 0.01). There was no significant difference in groups D1, B1, and B3 (*P* > 0.05), compared to group D2. The level of H_2_O_2_ increased significantly in group B1 and group B3 (*P* < 0.01).

#### 3.5.6. Changes of Activity of MDA

As shown in Figure 6(e), the activity of MDA increased significantly in groups M, B1, and B2, compared to group S (*P* < 0.01). In groups pretreated with high dose of Dex (group D2) and ARC239 (group B2), MDA decreased significantly compared to group M (*P* < 0.01). There was no significant difference in groups D1, B1, and B3 (*P* > 0.05), compared to group D2. The level of MDA increased significantly in group B1 and group B3 (*P* < 0.01).

## 4. Discussion

Acute kidney injury (AKI) is a severe complication following OLT. It is the major reason for early postoperation death and influences the prognosis and rehabilitation of patients. However the cause of AKI involves multifactors and the mechanisms contributing to this are still unclear [26, 27]. Our present study used an OALT model which simulates the key surgical procedures and pathophysiological processes that occur during liver transplantation, including hemodynamic changes, congestion, hypoxia, and hepatic ischemia-reperfusion injury [28–30]. The present study identified that OALT causes serious injury to the pathological structure in the kidney 8 h following the procedure [31]. The model was suitable to investigate the role of Nrf2 in kidney damage after liver transplantation and evaluate the protective effects of dexmedetomidine in this pathology.

Oxidative damage that is induced by reactive oxygen species (ROS) plays an important role in the pathogenesis of acute kidney injury [32]. Some researchers reported that, during the state of ischemia and hypoxia, the content of ATP in kidney cells is decreased, which causes calcium overload of cells and upregulation of hypoxanthine and xanthine oxidase. This can affect mitochondrial function and increase sympathetic tone by generation of large amounts of free radicals including oxygen free radicals and lipid free radicals [33, 34]. The free radicals are very active and increase levels of radicals by chain reaction [35]. The free radicals will attack cell membrane, protein, DNA or RNA, and extracellular matrix and change their structure and then cause multiple organ dysfunction involving kidney damage [36]. The latter can present as changes in kidney histology (cell degeneration) and renal failure (increasing Scr and BUN levels). The current study showed that the increases in ROS such as MDA, OH, and H_2_O_2_ were related to renal I/R damage. However the correlation between perioperative kidney injury and oxidative stress in liver transplantation remains unclear [35, 37].

Nrf2 is a transcription factor, which belongs to the cap “n” collar basic leucine zipper family. It is a major endogenous antioxidant and is distributed widely in terms of cells, tissues, and organisms. Under normal conditions, Nrf2 is repressed by its association with Keap1 which targets Nrf2 for ubiquitination and proteasomal degradation. Under conditions of chemical/oxidative stress, Nrf2 evades repression by Keap1 and accumulates within the nucleus, where it binds to antioxidant response elements and upregulates the expression of target genes harboring antioxidant response elements. It maintains homeostasis [38, 39]. Dexmedetomidine is a highly selective *α*_2_ adrenergic receptor agonist with sedative, analgesic, and sympatholytic properties [40]. Many studies have reported that pretreatment with dexmedetomidine can relieve the kidney I/R damage by inhibiting the secretion of inflammatory cytokines, decreasing oxidative stress, and decreasing the numbers of neutrophils in the kidney [30, 41]. In the current research, we provided evidence that dexmedetomidine regulates ROS and antioxidation through Nrf2 and may play an important role in preventing renal I/R damage. These protective effects were reversed by a nonspecific *α*_2_ receptor blocker and a specific *α*_2A_ receptor blocker.

## 5. Conclusions

In conclusion, we have found that administration of high dose dexmedetomidine was associated with a renal protective effect. We demonstrated that Nrf2 plays a critical role in decreasing OLT-induced AKI. Pretreatment with dexmedetomidine upregulated antioxidation and downregulated oxidation by increasing the expression of Nrf2, ultimately providing a protective effect against AKI after OLT. The increase in cells and immunofluorescence intensities with positive Nrf2 protein expression as shown in Figure 5 should support our notion that activation of Nrf2 may represent an important mechanism whereby dexmedetomidine inhibits post-OALT kidney injury. However, many questions remain including what dose of dexmedetomidine provides the best protection. Further research to explore this subject will be required.

## Figures and Tables

**Table 1:** The weights of rats and the times of anhepatic phase among 7 groups (the data were represented as mean ± standard deviation (SD), *n* = 8).

Groups	Weight (g)	Time of anhepatic phase (min)
Group S	241.3 ± 18.1	—
Group M	240.6 ± 15.0	19.9 ± 0.5
Group D1	234.1 ± 20.3	19.8 ± 0.8
Group D2	240.5 ± 16.8	20.0 ± 0.6
Group B1	243.4 ± 21.6	20.0 ± 0.7
Group B2	243.1 ± 17.5	20.1 ± 0.6
Group B3	239.9 ± 21.0	20.2 ± 0.5

## References

[1] Zhang Q., Lu M. (2005). Liver transplantation is the therapy of end stage liver diesase. *Guangdong Medicine*.

[2] Hei Z.-Q., Li X.-Y., Shen N., Pang H.-Y., Zhou S.-L., Guan J.-Q. (2008). Prognostic values of serum cystatin C and *β*2 microglobulin, urinary *β*2 microglobulin and N-acetyl-*β*-D-glucosaminidase in early acute renal failure after liver transplantation. *Chinese Medical Journal*.

[3] Pham P.-T. T., Pham P.-C. T., Wilkinson A. H. (2009). Management of renal dysfunction in the liver transplant recipient. *Current Opinion in Organ Transplantation*.

[4] Wei Y.-G., Li B., Yan L.-N. (2006). An analysis of risk factors leading to post-liver transplantation acute renal failure. *Chinese Journal of General Surgery*.

[5] Ojo A. O., Held P. J., Port F. K. (2003). Chronic renal failure after transplantation of a nonrenal organ. *The New England Journal of Medicine*.

[6] Afonso R. C., Hidalgo R., Zurstrassen M. P. V. C. (2008). Impact of renal failure on liver transplantation survival. *Transplantation Proceedings*.

[7] Moi P., Chan K., Asunis I., Cao A., Kan Y. W. (1994). Isolation of NF-E2-related factor 2 (Nrf2), a NF-E2-like basic leucine zipper transcriptional activator that binds to the tandem NF-E2/AP1 repeat of the beta-globin locus control region. *Proceedings of the National Academy of Sciences of the United States of America*.

[8] Magesh S., Chen Y., Hu L. (2012). Small molecule modulators of Keap1-Nrf2-ARE pathway as potential preventive and therapeutic agents. *Medicinal Research Reviews*.

[9] Park H.-M., Cho J.-M., Lee H.-R., Shim G.-S., Kwak M.-K. (2008). Renal protection by 3H-1,2-dithiole-3-thione against cisplatin through the Nrf2-antioxidant pathway. *Biochemical Pharmacology*.

[10] Yoon H.-Y., Kang N.-I., Lee H.-K., Jang K. Y., Park J.-W., Park B.-H. (2008). Sulforaphane protects kidneys against ischemia-reperfusion injury through induction of the Nrf2-dependent phase 2 enzyme. *Biochemical Pharmacology*.

[11] Liu M., Yao X. D., Li W. (2015). Nrf2 sensitizes prostate cancer cells to radiation via decreasing basal ROS levels. *BioFactors*.

[12] Sykiotis G. P., Bohmann D. (2008). Keap1/Nrf2 signaling regulates oxidative stress tolerance and lifespan in *Drosophila*. *Developmental Cell*.

[13] Civantos Calzada B., de Artiñano A. A. (2001). Alpha-adrenoceptor subtypes. *Pharmacological Research*.

[14] Jakob S. M., Ruokonen E., Grounds R. M. (2012). Dexmedetomidine vs midazolamor propofol for sedation during prolonged mechanical ventilation: two randomized controlled trials. *The Journal of the American Medical Association*.

[15] Ji F., Li Z., Nguyen H. (2013). Perioperative dexmedetomidine improves outcomes of cardiac surgery. *Circulation*.

[16] Bell M. T., Puskas F., Bennett D. T. (2014). Dexmedetomidine, an *α*-2a adrenergic agonist, promotes ischemic tolerance in a murine model of spinal cord ischemia-reperfusion. *Journal of Thoracic and Cardiovascular Surgery*.

[17] Wanga Z., Koub D., Lic Z., Hea Y., Yua W., Du H. (2014). Effects of propofol-dexmedetomidine combination on ischemia reperfusion-induced cerebral injury. *NeuroRehabilitation*.

[18] Chi X., Zhang A., Luo G. (2013). Knockdown of myeloid differentiation protein-2 reduces acute lung injury following orthotopic autologous liver transplantation in a rat model. *Pulmonary Pharmacology and Therapeutics*.

[19] Luo C., Yuan D., Li X. (2015). Propofol attenuated acute kidney injury after orthotopic liver transplantation via inhibiting gap junction composed of connexin 32. *Anesthesiology*.

[20] He M., Pan H., Chang R. C.-C., So K.-F., Brecha N. C., Pu M. (2014). Activation of the Nrf2/HO-1 antioxidant pathway contributes to the protective effects of lycium barbarum polysaccharides in the rodent retina after ischemia-reperfusion-induced damage. *PLoS ONE*.

[21] Oliveira T. M., Sakai V. T., Machado M. A. A. M. (2008). COX-2 inhibition decreases VEGF expression and alveolar bone loss during the progression of experimental periodontitis in rats. *Journal of Periodontology*.

[22] Peng X., Tang J., Wu Y., Yang B., Hu J. (2014). Novel method for ANA quantitation using IIF imaging system. *Journal of Immunological Methods*.

[23] Jiang G., Liu X., Wang M., Chen H., Chen Z., Qiu T. (2015). Oxymatrine ameliorates renal ischemia-reperfusion injury from oxidative stress through Nrf2/HO-1 pathway. *Acta Cirurgica Brasileira*.

[24] Wang C., Blough E., Arvapalli R. (2015). Acetaminophen attenuates glomerulosclerosis in obese Zucker rats via reactive oxygen species/p38MAPK signaling pathways. *Free Radical Biology and Medicine*.

[25] Queisser N., Oteiza P. I., Link S., Hey V., Stopper H., Schupp N. (2014). Aldosterone activates transcription factor Nrf2 in kidney cells both in vitro and in vivo. *Antioxidants & Redox Signaling*.

[26] Yalavarthy R., Edelstein C. L., Teitelbaum I. (2007). Acute renal failure and chronic kidney disease following liver transplantation. *Hemodialysis International*.

[27] Leithead J. A., Ferguson J. W., Bates C. M., Davidson J. S., Simpson K. J., Hayes P. C. (2011). Chronic kidney disease after liver transplantation for acute liver failure is not associated with perioperative renal dysfunction. *American Journal of Transplantation*.

[28] Zhao H.-F., Zhang G.-W., Zhou J., Lin J.-H., Cui Z.-L., Li X.-H. (2009). Biliary tract injury caused by different relative warm ischemia time in liver transplantation in rats. *Hepatobiliary and Pancreatic Diseases International*.

[29] Jin C., Zhang P.-J., Wu X.-M. (2009). Impact of hypoxic preconditioning on apoptosis and its possible mechanism in orthotopic liver autotransplantation in rats. *Hepatobiliary and Pancreatic Diseases International*.

[30] Yuan C.-H., Xiu D.-R., Li Z.-F. (2012). Analysis of risk factors for acute renal failure in the early stage after liver transplantation. *Ogran Transplantation*.

[31] Guo-Liang S., Xin-Jin C., Yi J., Zi-Qing H. (2011). Acute kidney injury after orthotopic liver autotransplantation in SD rat model. *Journal of Sun Yat-Sen University(Medical Sciences)*.

[32] Heimlich J. B., Speed J. S., Bloom C. J., O'Connor P. M., Pollock J. S., Pollock D. M. (2015). ET-1 increases reactive oxygen species following hypoxia and high-salt diet in the mouse glomerulus. *Acta Physiologica*.

[33] Mendes-Braz M., Elias-Miró M., Kleuser B. (2014). The effects of glucose and lipids in steatotic and non-steatotic livers in conditions of partial hepatectomy under ischaemia-reperfusion. *Liver International*.

[34] Usta J., Hachem Y., El-Rifai O. (2013). Fragrance chemicals lyral and lilial decrease viability of HaCat cells' by increasing free radical production and lowering intracellular ATP level: protection by antioxidants. *Toxicology in Vitro*.

[35] Sung C.-C., Hsu Y.-C., Chen C.-C., Lin Y.-F., Wu C.-C. (2013). Oxidative stress and nucleic acid oxidation in patients with chronic kidney disease. *Oxidative Medicine and Cellular Longevity*.

[36] Cui J., Liu J., Wu S. (2013). Oxidative DNA damage is involved in ochratoxin A-induced G2 arrest through ataxia telangiectasia-mutated (ATM) pathways in human gastric epithelium GES-1 cells in vitro. *Archives of Toxicology*.

[37] Radak Z., Zhao Z., Koltai E., Ohno H., Atalay M. (2013). Oxygen consumption and usage during physical exercise: the balance between oxidative stress and ROS-dependent adaptive signaling. *Antioxidants and Redox Signaling*.

[38] Shelton L. M., Park B. K., Copple I. M. (2013). Role of Nrf2 in protection against acute kidney injury. *Kidney International*.

[39] Ke B., Shen X.-D., Zhang Y. (2013). KEAP1-NRF2 complex in ischemia-induced hepatocellular damage of mouse liver transplants. *Journal of Hepatology*.

[40] Kunisawa T. (2011). Dexmedetomidine hydrochloride as a long-term sedative. *Therapeutics and Clinical Risk Management*.

[41] Ueki M., Kawasaki T., Habe K., Hamada K., Kawasaki C., Sata T. (2014). The effects of dexmedetomidine on inflammatory mediators after cardiopulmonary bypass. *Anaesthesia*.

